# Rare types of congenital adrenal hyperplasia: report of five children with 11β-hydroxylase deficiency including pathogenic and novel *CYP11B1* variants

**DOI:** 10.20945/2359-4292-2026-0073

**Published:** 2026-07-03

**Authors:** Anju Bala, Sayan Banerjee, Arun George, Priyanka Srivastava, Mohit Kumar, Neha KC, Jaivinder Yadav, Rakesh Kumar, Devi Dayal

**Affiliations:** 1 Endocrinology and Diabetes Unit, Department of Pediatrics, Postgraduate Institute of Medical Education and Research, Chandigarh, India

**Keywords:** 11-beta hydroxylase deficiency, hormonal profile, hypertension, peripheral precocious puberty, genetic variations, India

## Abstract

11β-hydroxylasedeficiency (11β-OHD) is a rare form of congenital adrenal hyperplasia caused by biallelic pathogenic variants in the *CYP11B1* gene. It leads to impaired cortisol synthesis, resulting in increased adrenocorticotropic hormone stimulation and consequent accumulation of steroid precursors, which are diverted to androgen synthesis. In addition, the accumulation of 11-deoxycorticosterone, which is a potent mineralocorticoid, causes hyporeninemic hypokalemic hypertension. We report the clinical, hormonal, and genetic profiles of five children with 11β-OHD, emphasising phenotypic variability, a median 2-year diagnostic delay, the crucial role of hormonal profile in diagnosis, and management challenges, including post-treatment central precocious puberty. Two novel *CYP11B1* variants were identified in two unrelated patients. Hydrocortisone replacement resolved hypertension in only one of the three hypertensive patients; others required spironolactone. Early differentiation of 11β-OHD from 21-hydroxylase deficiency is critical to prevent hypertension-related morbidity.

## INTRODUCTION

Congenital adrenal hyperplasia (CAH) refers to a group of autosomal recessive genetic endocrine disorders in children caused by variants in the genes encoding the various enzymes involved in adrenal steroidogenesis. Depending on the specific enzymatic defect, the synthesis of glucocorticoids, mineralocorticoids, and sex steroids may be impaired, resulting in varied manifestations ranging from genital ambiguity to potentially life-threatening adrenal insufficiency (^1^). The most common form of CAH is 21-hydroxylase deficiency (21-OHD), which is caused by variants in the *CYP21A2* gene, accounting for ~95% of cases. Other forms such as 11β-hydroxylase deficiency (11β-OHD) due to *CYP11B1* gene variants, 3β-hydroxysteroid dehydrogenase type 2 deficiency (*HSD3B2* gene variants), steroid 17α-hydroxylase/17,20 lyase (*CYP17A1* gene variants), P450 cholesterol side-chain cleavage enzyme (*CYP11A1* gene variants), or the steroidogenic acute regulatory protein (*STAR* gene variants), are rare and together account for ~5% cases (^1^). The prevalence of non-21-OHD forms of CAH varies across geographical regions. While 11β-OHD is generally considered the second most common form of CAH, this is not the case in certain countries. For example, 17-hydroxylase deficiency in Brazil and StAR protein deficiency in Japan, China, and India are the most common 21-OHD variants of CAH (^1^-^5^).

In countries with well-established newborn screening (NBS) programs that employ 17-hydroxyprogesterone (17-OHP) measurements, the early detection of most classic 21-OHD cases and some rare variants has significantly improved long-term outcomes. However, in countries with limited or no nationwide NBS programs, such as India, timely diagnosis of CAH remains a challenge (^6^). Late diagnoses are common even for classic CAH; in a north Indian center, 75% were diagnosed only after they presented in adrenal crisis (^7^). Diagnosing rarer CAH variants is even more difficult due to variable increases in 17-OHP concentrations and significant clinical heterogeneity (^8^). Of all the non-21-OHD variants of CAH, 11β-OHD is often challenging to diagnose due to its distinct effects on adrenal hormone synthesis and consequent clinical manifestations (^8^). The enzyme 11β-OH mediates the conversion of 11-deoxycorticosterone (DOC) to corticosterone and 11-deoxycortisol to cortisol. 11β-OHD, therefore, results in the accumulation of 11-DOC, which is a potent mineralocorticoid and causes hyporeninemic hypokalemic hypertension. The diversion of steroid precursors to androgen synthesis produces features such as ambiguous genitalia in female infants, and virilization and precocious puberty during childhood mimicking simple virilizing CAH (^9^). During infancy, cortisol deficiency may present as a salt-wasting crisis similar to classic 21-OHD (^1^,^8^). Although hypertension is a distinguishing feature, it may develop late in the disease course (^10^). Due to such diagnostic challenges, children with 11β-OHD are often misclassified as 21-OHD initially (^8^).

Over the last few years, the detection and outcome of children with 11β-OHD have improved due to the wider availability of hormonal profiling by liquid chromatography tandem mass spectrometry (LC-MS/MS), as it simultaneously measures different adrenal steroids and their precursors (^11^). Additionally, the reduction in the costs of genetic analysis has enabled the confirmation of diagnosis in previously unresolved cases (^12^). The distribution of pathogenic variants also differs among different ethnicities. Of the more than 200 *CYP11B1* gene variants reported to date, most are missense or nonsense variants, occurring predominantly in exons 2, 6, 7, and 8. Worldwide, the most common variant identified is the p.R448H in exon 8; this is especially recurrent in Moroccan Jews. Other variants, such as p.R454C and p.R448P, are prevalent in China and Saudi Arabia, respectively, whereas p.Q356X is found in the Tunisian population and African Americans (^13^). At our tertiary care pediatric hospital in North India, we have been following a large cohort of children with CAH, most of whom are due to 21-OHD. This clinical brief describes our experience of 11β-OHD, focusing on varied clinical presentations, diagnostic delays, blood hormone profiling, genetic analysis, including two novel variants, and management strategies.

## CASE SERIES

The Department Review Board (DRB-37-25) approved this retrospective study, and informed consent was obtained from the caregivers of all the patients for publication. Five patients [four boys, median age (IQR) 6.5 (3-6.8 years)] were diagnosed with 11β-OHD out of our 20-year cohort of approximately 60 patients with CAH. Boys presented with peripheral precocious puberty (PPP), skin hyperpigmentation, and hypertension, while the girl presented with clitoromegaly. One patient (Case 2) was born of third-degree consanguinity; his elder brother also had precocious puberty and short stature. There was no history of consanguinity in the other four cases. Clinical and laboratory workup revealed tall stature and advanced bone age (BA) in all, metabolic alkalosis in four, hypertension, and hypokalaemia in three children (**Table 1**). Cases 1, 2, and 3 all had hypertension, metabolic alkalosis, and hypokalemia at presentation. Case 2, presented with hypertensive emergency, left ventricular hypertrophy (LVH), and testicular adrenal rests on ultrasound. Case 4 presented at 2 years of age with a history of ambiguous genitalia (clitoromegaly) noticed from 5 months of age. At her presentation, metabolic alkalosis was the only documented abnormality, without evidence of hypertension or hypokalemia. Four patients were evaluated using a hormonal profile by LC-MS/MS, which showed elevated levels of 11-deoxycortisol (median: 702 nmol/L), 11-DOC (median: 44.96 nmol/L), 17-OHP (median: 14.25 nmol/L), and androstenedione (median: 47.83 nmol/L) (**Table 1**).

**Table 1:** Clinical, anthropometric, and biochemical parameters of patients

Variable	Case 1	Case 2	Case 3	Case 4	Case 5
**At initial Presentation**					
Age at presentation (years)/Sex	6.6/M	6.5/M	7/M	2/F	4.5/M
Height (SDS)	3.74	5.38	4.02	-0.31	3.44
Weight (SDS)	1.8	2.92	2.69	0.37	2.04
BMI (SDS)	0.1	0.64	1.51	0.77	0.01
Bone age (years)	13	12	14	4	8
Blood pressure	Stage 1 HTN	Stage 2 HTN	Stage 2 HTN	No HTN	No HTN
External genitalia findings	SPL 6 cm, TV 3 cc, PH 4	SPL 9.5 cm, TV 3 cc, PH 4	SPL 8 cm, TV 4 cc, PH 4	Clitoral length 2.5 cm	SPL 6.6 cm, TV 4 cc, PH 3
ACTH (pmol/L)/(pg/mL)	212.3 /965	92.8/422	440/2000	19.5/88.5	215.6/980
Cortisol (nmol/L)/(µg/dL)	92.9/3.37	48.56/1.76	61.9/2.24	94.5/3.43	69.36/2.51
Basal LH (mIU/mL)	0.41	0.27	0.30	0.28	0.10
Basal FSH (mIU/mL)	0.48	0.25	0.30	0.30	0.10
Serum potassium (mmol/L)	3	3	3.4	3.9	3.7
Metabolic alkalosis	Yes	Yes	Yes	Yes	No
17-OHP (nmol/L)/(ng/dL)	10.42/344	20.33/671	37.26/1230	31.51/1040	14.27/471
Testosterone (nmol/L) /(ng/dL)	7.30/210.56	13.15/379	7.40/213.36	2.77/80	4.70/471
Androstenedione (nmol/L) /(µg/L)	67.38/19.30	189/54.20	7.56/2.16	47.83/13.70	-
DHEAS (nmol/L)/(µg/dL)	5.69/210	6.31/233	2.62/97	5.01/185	2.34/86.7
11-Deoxycortisol (nmol/L) /(µg/L)	363.60/120	996.87/329	790.83/216	748.41/247	-
11-Deoxycorticosterone (nmol/L)/(µg/L)	21.63/7.14	45.75/15.10	44.23/14.6	136.35/45	-
CPP post-treatment (duration)	Yes (6 months)	Yes (3 months)	Yes (9 months)	No	Yes (6 months)
TV at diagnosis of CPP	5cc	5cc	6cc	-	6cc
LH (mIU/mL) at diagnosis of CPP	2.40	1.82	3.38	-	2.91
**At the last follow-up**					
Age at last follow-up (years)	7.6	7.5	9	3.5	18
Height (SDS)	3.10	2.97	1.95	-0.65	-3.03
BMI (SDS)	0.48	1.05	1.25	0.75	-1.09
Therapy at the last follow-up	Hydrocortisone, Leuprolide 11.25 mg	Hydrocortisone, Spironolactone, Leuprolide 11.25 mg	Hydrocortisone, Spironolactone, Leuprolide 11.25 mg	Hydrocortisone	Dexamethasone 0.5 mg

ACTH: Adrenocorticotropic hormone; SDS: Standard deviation scores; BMI: Body mass Index; LH: Luteinizing hormone; FSH: Follicle-stimulating hormone; DHEAS: Dehydroepiandrosterone sulfate; 11β-OHD: 11β-hydroxylase deficiency; 17-OHP: 17-hydroxyprogesterone; CPP: Central precocious puberty; GnRH: Gonadotropin-releasing hormone; SPL: Stretched penile length; TV: Testicular volume; PH: Pubic hair; HTN: Hypertension.

**Note: Reference range for parameters in SI units:** 17-OHP: 0.94-6.56 nmol/L (male), 0.30-2.42 nmol/L (female); Androstenedione: 0.27-1.74 nmol/L, 11-Deoxycortisol: 0.60-7.57 nmol/L, 11-Deoxycorticosterone: 0.06-1.03 nmol/L, ACTH: 1.58-13.93 pmol/L, Cortisol: 171-536 nmol/L, Testosterone: <0.34 nmol/L, DHEAS: 0.013-0.527 nmol/L (1-4yr), 0.076-2.314 nmol/L (5-9yr) **Reference range for parameters in conventional units:** 17-OHP: 31-217 ng/dL (male), 10-80 ng/dL (female), Androstenedione: 0.08-0.50 µg/L, 11-Deoxycortisol: 20-216 µg/L, 11-Deoxycorticosterone: 0.02-0.34 µg/L, ACTH: 7.2-63.2 pg/mL, Cortisol: 6.2-19.4 µg/dL, Testosterone: <9.8 ng/dL, DHEAS: 0.48-19.5 µg/dL (1-4 yr), 2.8-85.4 µg/dL (5-9 yr).

**Figure 1:**
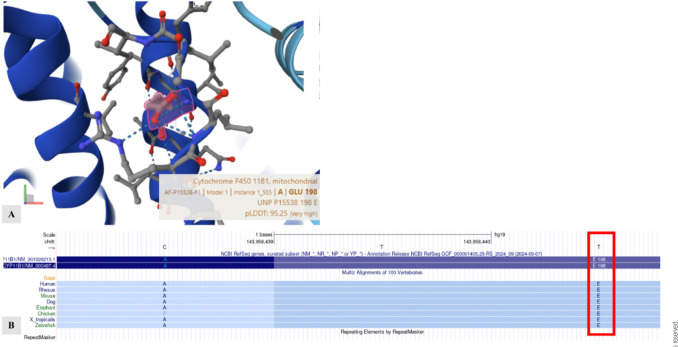
**(A)** AlphaFold-predicted structure of wild-type *CYP11B1* showing the spatial localization of residue Glu198. The three-dimensional structure of human *CYP11B1* (UniProt P15538) was obtained from the AlphaFold Protein Structure Database. Residue Glu198 (red sticks) is highlighted within the conserved cytochrome P450 fold. As the c.594A>G variant is synonymous (p.E198=), no alteration in protein conformation is observed, suggesting that pathogenicity is likely mediated through RNA-level regulatory mechanisms rather than structural changes. **(B)** Multiple sequence alignment of CYP11B1 protein sequences across vertebrate species.

Molecular analysis of the *CYP11B1* gene by next-generation sequencing (NGS) using clinical exome sequencing identified five distinct variants in the five unrelated patients, including two novel variants (**Table 2**). All patients were homozygous for variants inherited in an autosomal recessive manner. Among these, one variant was classified as pathogenic, three as likely pathogenic, and one as a variant of uncertain significance (VUS) based on the standard guidelines of the American College of Medical Genetics and Genomics (ACMG) classification (^14^). The variants comprised two nonsense (p.Gln338Ter, p.Tyr266Ter), one missense variant (p.Cys450Tyr), one synonymous variant (p.Glu198=), and one homozygous contiguous gene deletion of 37.21 kb. Case 1 harbored a novel (ClinVar Accession number: SCV006555343) homozygous synonymous variant c.594A>G (p.Glu198=) in exon 3, classified as a VUS due to the lack of functional studies. Structural visualization using AlphaFold demonstrated that Glu198 localizes within a conserved region of the *CYP11B1* protein (**Figure 1A**). As the c.594A>G variant does not alter the amino-acid sequence, no conformational differences were observed between wild-type and variant proteins (**Figure 1B**). Case 5 also exhibited a novel (Accession number: SCV006555344) homozygous likely pathogenic missense variant c.1349G>A (p.Cys450Tyr) in exon 8. In silico parameters suggested damaging and deleterious effects. All variants were considered definitely homozygous based on the molecular tools used. However, segregation analysis could not be performed due to financial constraints. The ACMG classification of the variants is given in **Table 2**.

**Table 2:** Details of molecular analysis of the *CYP11B1* gene in the studied patients

Case	1	2	3	4	5
Location	Exon 3	Exon 6	Exon 4	Chr 8	Exon 8
Variant	c.594A>G	c.1012C>T	c.798C>G	g.(?*142875702*)(142912910_?) del	c.1349G>A
Amino Acid Change	p.Glu198=	p.Gln338Ter	p.Tyr266Ter	-	p.Cys450Tyr
Zygosity	Homozygous	Homozygous	Homozygous	Homozygous	Homozygous
Inheritance	AR	AR	AR	AR	AR
Classification	VUS	Pathogenic	Likely Pathogenic	Pathogenic	Likely Pathogenic
Novel/Reported	Novel	Reported	Reported	Reported	Novel
ACMG Criteria	PM2, PP3	PVS1, PM2, PP5	PVS1, PM2, PP3	PVS, PM2, PP4	PM1, PM2, PP3, PP4
Type of Mutation	Synonymous	Nonsense	Nonsense	Homozygous deletion of 37.21KB	Missense

AR: autosomal recessive; ACMG: American College of Medical Genetics and Genomics; *CYP11B1:* Cytochrome P450 family 11 subfamily B member 1; VUS: variant of uncertain significance; 11β-OHD: 11β-hydroxylase deficiency PVS1: pathogenic very strong 1; PM2: pathogenic moderate 2; PP3: pathogenic supporting 3; PP4: pathogenic supporting 4; PP5: pathogenic supporting 5.

There was a median of 2 (IQR-0.5) years delay from the onset of symptoms to definitive diagnosis. Cases 1, 2, and 3 were diagnosed with 11β-OHD at initial presentation based on hypertension, hypokalemia, and metabolic alkalosis. The initial clinical diagnoses were simple virilizing CAH (21-OHD) in two patients (cases 4 and 5); hormonal profiling helped reach a definitive diagnosis of 11β-OHD in one of these (**Table 1**). In case 5, the definitive diagnosis was made only after molecular analysis at age 18 years.

All children received hydrocortisone (10-15 mg/m^2^/day); case 5 also received fludrocortisone until a confirmatory diagnosis was made and was shifted to dexamethasone at the age of 14 years. Two (cases 2 and 3) of the three children with hypertension at diagnosis required initial management with labetalol infusion. Only one child (case 1) showed resolution of hypertension with hydrocortisone replacement; the other two required the addition of spironolactone for control of hypertension (**Table 1**). All four boys developed central precocious puberty (CPP) within 3-9 months after initiation of glucocorticoid therapy. The diagnosis of CPP was based on an increase in testicular volume (>4 cc) and luteinizing hormone (LH) levels from baseline (**Table 1**). All four were started on leuprolide acetate 11.25 mg intramuscularly every 3 months. Cases 1, 2, and 3 are currently receiving leuprolide and have not shown further progression of puberty, while case 5 (current age 18 years) has completed puberty following leuprolide discontinuation at age 12 years. The median follow-up duration was 2.5 years (1.65-8.25 years).

## DISCUSSION

11β-OHD impairs the conversion of 11-DOC to corticosterone and 11-deoxycortisol to cortisol. Cortisol deficiency leads to an increase in adrenocorticotropic hormone (ACTH), which causes the accumulation of steroid precursors such as 11-DOC and 17-OHP, which are diverted to androgen synthesis (^1^). Girls present with virilised genitalia at birth, but boys often present later with PPP and hypertension (^1^,^8^). Hypertension and hypokalemia occur due to chronic elevation of 11-DOC, which exerts potent mineralocorticoid effects (^1^). When present, hyporeninemic hypertension helps differentiate 11β-OHD from other CAH variants (^10^). However, not all patients present with hypertension. The onset and prevalence of hypertension are variable and usually attributed to the degree of increase in aldosterone precursors (^10^). However, hypertension in patients with 11β-OHD shows no direct correlation with 11-DOC levels or the degree of virilization (^8^). One proposed mechanism that warrants further exploration is genotype-dependent residual 11-β hydroxylase activity, as severe variants have been reported to be associated with worse hypertension (^15^). Two of our patients did not have hypertension at any time during their clinical course. When hypertension is a presenting feature, adrenal tumors and apparent mineralocorticoid excess also need consideration initially (^16^).

Based on the most commonly employed laboratory test for diagnosing CAH, i.e., 17-OHP, 11β-OHD is often misclassified as 21-OHD, leading to its underdiagnosis, even during NBS for CAH (^17^). In our patients, the 17-OHP elevations were also variable; case 5 was thus misdiagnosed as simple virilising 21-OHD for 14 years in the absence of hypertension or hypokalemia. Although a borderline elevation in 17-OHP warrants measurement of 11-deoxycortisol, the diagnostic cutoff for 11β-OHD is not defined (^17^). In countries where NBS for 21-OHD is performed, some false-positive results may still be 11β-OHD cases. A careful further evaluation of borderline 17-OHP values on NBS is therefore warranted. A previous study confirmed a diagnosis of 11β-OHD in 1% of their 133 patients with a previous diagnosis of 21-OHD (^17^). In such unsolved cases, hormonal profiling helps identify the enzymatic defect by estimating intermediate molecules in the steroidogenic pathway (^11^,^17^). In addition to 11-deoxycortisol, 11-DOC, and androstenedione, 21-deoxycortisol, a derivative of 17-OHP via 11-hydroxylase action, may serve as an alternative steroid marker for identifying 21-OHD subtypes, including heterozygotes, and may aid in distinguishing 11β-OHD (^17^). In our series, the 17-OHP levels of 31.47 nmol/L, without hypertension in case 4, pointed toward 21-OHD; however, the simultaneous elevations of 11-DOC and 11-deoxycortisol in the hormonal profile suggested 11β-OHD. In case 3, although 17-OHP levels were >30 nmol/L, the presentation with grade 2 hypertension provided an initial clue to the diagnosis. Thus, hormonal profiling further helps differentiate 11β-OHD from 21-OHD when initial clinical clues such as hypertension, hypokalemia, and metabolic alkalosis are absent. Molecular analysis subsequently confirms the enzymatic defect and is critical for genetic counseling and risk assessment in future pregnancies (^17^).

Our study highlights a heterogeneous variant spectrum in 11β-OHD, with two novel variants identified in five patients. A homozygous synonymous variant, c.594A>G (p.Glu198=) in exon 3, was classified as a VUS due to the absence of functional validation. Synonymous variants, however, can disrupt protein function by altering splicing, mRNA stability, translation efficiency, or transcription factor binding (^18^). Case 5 harbored a homozygous missense variant, c.1349G>A (p.Cys450Tyr) in exon 8, affecting a highly conserved residue in *CYP11B1* and predicted to impair protein structure and function. Prior studies indicate variant clustering in exons 2, 6, 7, and 8, consistent with known genetic hotspots. A nonsense variant, c.798C>G (p.Tyr266Ter) in exon 4, introduces a premature stop codon, producing a truncated, nonfunctional protein, as reported in ClinVar. The nonsense variant p.Gln338Ter in case 2, previously described in the Turkish population, results in complete enzyme inactivation (^19^). Case 4 exhibited a large 37.2 kb homozygous contiguous gene deletion encompassing *CYP11B1* and *CYP11B2*, consistent with earlier reports of locus loss or fusion leading to 11β-OHD (^20^). Our data provide further support for regional genetic heterogeneity in patients with 11β-OHD from India (^21^).

The prevalence of hypertension in patients with 11β-OHD varies between 30 and 66% and correlates significantly with older age at diagnosis (^15^,^22^,^23^). It often causes significant acute and chronic morbidity; case 2 presented with a hypertensive emergency, while his elder brother showed LVH due to chronic hypertension. If hypertension persists despite optimal glucocorticoid therapy, the addition of spironolactone, amiloride, a calcium channel blocker, or even bilateral adrenalectomy should be considered (^15^). Poorly controlled hypertension and glucocorticoid excess in 11β-OHD may cause cardiometabolic complications, including end-organ damage such as LVH, retinopathy, nephropathy, and cerebrovascular events (^8^,^15^).

In our series, four patients developed CPP after treatment initiation, unlike other forms of CAH, where CPP may be a presenting feature (^24^). This likely results from the withdrawal of prolonged gonadotropin-independent androgen exposure, which subsequently triggers activation of the hypothalamic-pituitary-gonadal axis (^25^). Intratesticular adrenal cell rests, as seen in case 2, may reflect disease severity and delayed diagnosis, and contribute to male infertility by compressing the seminiferous tubules and causing obstructive azoospermia (^8^). Advanced BA, seen in all our patients, also reflects chronic androgen exposure due to delayed diagnosis and carries a poor adult height prognosis (^24^,^25^).

## CONCLUSIONS

11β-OHD is a rare form of CAH with high phenotypic variability, leading to diagnostic delays. Borderline 17-OHP elevations in suspected CAH cases should prompt evaluation for 11β-OHD. Hormonal profiling helps to differentiate this rarer form from 21-OHD. The two novel variants identified in our patients expand the known genetic spectrum of the *CYP11B1* in the Indian population.

## Data Availability

datasets related to this article will be avail-able upon request to the corresponding author.
